# Lipid Bilayer Composition Affects Transmembrane Protein Orientation and Function

**DOI:** 10.1155/2011/208457

**Published:** 2011-01-26

**Authors:** Katie D. Hickey, Mary M. Buhr

**Affiliations:** Department of Animal and Poultry Science, University of Guelph, Guelph, ON, Canada N1G 2W1

## Abstract

Sperm membranes change in structure and composition upon ejaculation to undergo capacitation, a molecular transformation which enables spermatozoa to undergo the acrosome reaction and be capable of fertilization. Changes to the membrane environment including lipid composition, specifically lipid microdomains, may be responsible for enabling capacitation. To study the effect of lipid environment on proteins, liposomes were created using lipids extracted from bull sperm membranes, with or without a protein (Na^+^ K^+^-ATPase or *α*-amylase). Protein incorporation, function, and orientation were determined. Fluorescence resonance energy transfer (FRET) confirmed protein inclusion in the lipid bilayer, and protein function was confirmed using a colourometric assay of phosphate production from ATP cleavage. In the native lipid liposomes, ATPase was oriented with the *β* subunit facing the outer leaflet, while changing the lipid composition to 50% native lipids and 50% exogenous lipids significantly altered this orientation of Na^+^ K^+^-ATPase within the membranes.

## 1. Introduction

Lipid rafts contain lipids, glycosylphosphatidylinositol- (GPI-) anchored proteins and various signal transduction proteins [1, 2] that engage the microdomain in cell signaling and cell adhesion events [3]. Capacitation is a process whereby spermatozoa gain the ability to fertilize through a series of membrane changes including a reorganization of membrane lipids and proteins [4, 5]. Lipid rafts in head membranes of uncapacitated sperm are uniformly distributed but become restricted to the periacrosomal region during capacitation [6], suggesting rafts are associated with capacitation. One particular protein involved in cell signaling during capacitation, Na^+^ K^+^-ATPase [7], is extremely sensitive to its lipid environment and its function is uniquely damaged when sperm are cryopreserved [8], a process that also reduces fertility. Loss of Na^+^ K^+^-ATPase function postthaw, whether caused by cryopreservation-induced direct damage to the protein and/or alterations in the protein's lipid environment [9], could cause the loss of fertility. Na^+^ K^+^-ATPase is a ubiquitous transmembrane protein consisting of alpha (*α*) and beta (*β*) subunits [10, 11], which is well known to function as an ion-transporting enzyme [12], but also has been recently shown to act as a transmembrane signalling molecule in somatic cells [13] and even more recently in sperm [7]. Although Na^+^ K^+^-ATPase is not associated with lipid rafts in brain synaptosomes [14], MDCK cells, or enterocytes [15], it is enriched in lipid raft fractions of gastric luminal cells [16], hepatocytes [17], renal epithelial [15], outer medulla kidney, and cardiac cells [18]. Interestingly, Na^+^ K^+^-ATPase is stimulated in muscle fibers and fish gill epithelium by a relocation which is mediated by lipid rafts [19, 20]. The specific effects of lipid environment on Na^+^ K^+^-ATPase in sperm membranes is unknown. 

Liposomes provide a model membrane system to study protein function [21, 22], facilitating assessment of integral membrane proteins without interference from other cell components that may obscure results [23]. Many proteoliposome studies attempt to reproduce a typical membrane by mixing a few commercial lipids as a base composition [24–26], but, logically, using extracted endogenous membrane phospholipids would even more closely mimic the native lipid bilayer including lipid rafts. Functional protein reconstitution into vesicles is dependent on method of preparation and lipid composition of the vesicles, necessitating determination of degree of incorporation of a protein into the liposomes before detailed protein function can be reliably and repeatedly evaluated. We hypothesized that lipid composition would affect Na^+^ K^+^-ATPase insertion into liposomes, and therefore measured incorporation, function and orientation of Na^+^ K^+^-ATPase in liposomes made from lipids extracted from the head plasma membrane (HPM) of bull sperm, and then altered the lipid composition of the proteoliposomes to determine the effect of lipid composition on the nature of the incorporation of Na^+^ K^+^-ATPase into lipid vesicles.

## 2. Materials and Methods

### 2.1. Materials

Adenosine 5′-triphosphate (ATP), sodium chloride (NaCl), HEPES, ouabain octahydrate, *α*-amylase (Type II-A from Bacillus species), bovine serum albumin (BSA), dog kidney Na^+^ K^+^-ATPase, osmium tetroxide, imidazole, ammonium molybdate L-Histidine, carbonyl cyanide 3-chlorophenylhydrazone (CCCP), valinomycin, phosphatidylcholine (PC; 16 : 0, 18 : 0, 18 : 1, and 18 : 2), phosphatidylethanolamine (PE), sphingomyelin (Sph; 18 : 0), and phosphatidylserine (PS; 16 : 0) were purchased from Sigma-Aldrich (Mississauga, ON, Canada). N-(dimethylaminonaphthalene-1-sulfonyl)-1,2-dihexadecanoyl-sn-glycero-3-phosphoethanolamine, triethylammonium salt (dansyl DHPE) was from Molecular Probes. Thermo Fisher Scientific was the supplier for chloroform, methanol, ascorbic acid, hydrochloric acid (HCl), sodium citrate dihydrate, sodium arsenite, and glacial acetic acid. Phosphatidylcholine (20 : 4), Sph (16 : 0), PS (18 : 0 and 18 : 2), and phosphatidylinositol (PI; 18 : 1) were purchased from Avanti (Alabaster, AL), and PC (22 : 6) and PI (16 : 0) were from Matreya (Pleasant Gap, PA).

### 2.2. Lipid Preparation

Lipids were extracted from the head plasma membrane (HPM) of whole bull sperm (33 ejaculates from 14 different bulls) using established methods [8, 9], pooled, dried, weighed, resolubilized in chloroform methanol (2 : 1, v/v), divided into equal aliquots containing 1.5 mg lipids, and stored at −70°C under nitrogen gas to provide identical lipid compositions for all liposomes.

### 2.3. Protein Preparation

Dog kidney Na^+^ K^+^-ATPase was purified using an ouabain affinity column [27], freeze dried and stored at −20°C. Freeze-dried samples were resuspended in HEPES-buffered saline (HBS; 150 mM NaCl; 8 mM HEPES; pH 7.2), pooled and re-aliquoted to provide identical working samples. Aliquots were refrozen under N_2_ at −70°C to preserve protein function. BSA and *α*-amylase were diluted in HBS to the desired concentration and used immediately.

### 2.4. Liposome and Proteoliposome Preparation

One aliquot of stored lipids was warmed to room temperature and dried (stream of N_2_ gas to visible dryness then 1 hr vacuum desiccation). Dried lipids were solubilized by incubation in HBS (0.75 mg lipid/mL; 60°C, 60 seconds), then vortexing (30 seconds) with scraping and further incubation (60°C, 30 seconds). Vortexing and incubation were repeated thrice and the resultant lipid suspension was either used for liposome preparation or placed on ice to cool while the protein was prepared. 

Protein (purified Na^+^ K^+^-ATPase, *α*-amylase, BSA) was diluted in ice-cold HBS (final concentration, 0.075 or 0.15 mg protein/mL), added to the cooled lipid suspension and vortexed (15 seconds). The resultant lipid protein ratio was 20 : 1 or 10 : 1 (w/w) in 3.0 mL (0.5 mg lipid/mL and 0.025 or 0.05 mg protein/mL). 

Liposomes or proteoliposomes were created using high pressure nitrogen filtration, which was the best of five methods for consistency of producing small, unilamellar liposomes using bull sperm HPM lipids. The lipid or lipid/protein suspension was forced through a 0.1 *μ*m Nuclepore Polycarbonate Membrane (Whatman Inc. Clifton, New Jersey, USA) using high-pressure nitrogen gas (35 kg/cm). Resulting liposomes or proteoliposomes were maintained under N_2_ gas until measurements were taken (within 30 minutes). 

### 2.5. Determination of Proteoliposome Properties

#### 2.5.1. Size

**Dynamic Light Scattering (DLS)**

Proteoliposome size was assessed by DLS with a Malvern Microfluidizer, model M-110-S (Malvern Instruments, Herrenberg, Germany), which provided six independent readings that were then converted by an internal algorithm into liposome diameter [28].

**Scanning Electron Microscopy (SEM)**

Proteoliposomes were settled on polished carbon planchettes, fixed (2 hours, 0.5% (w/v) OsO_4_ in 0.05 M veronal acetate and 0.2 M imidazole), rinsed (3x, 0.2 M imidazole), and then dehydrated in increasing concentrations of ethanol (50%, 70%, 90%, then 3x in 100%; [29]). Ethanol was removed by critical point drying with CO_2_. Planchettes were made conductive by sputter coating with Argon to 15 nm thick using Emscope K550 sputter coater, (Ashford, Kent, UK) and were viewed using a Hitachi S-570 Scanning Electron Microscope (Tokyo, Japan). Micrographs were captured using Quartz PCI software (Quartz Imaging Corp. Vancouver, Canada).

### 2.6. Protein Incorporation

Fluorescence resonance energy transfer (FRET) tested incorporation of protein into the bilayer, assessing the fluorescence emission of the FRET probe N-(dimethylaminonaphthalene-1-sulfonyl)-1,2-dihexadecanoyl-sn-glycero-3-phosphoethanolamine, triethylammonium salt (dansyl DHPE) with a spectrofluorometer (Photon Technology International, model A-1010, London, ON, Canada). Excitation scans first established optimal excitation and emission wavelengths for tryptophan and dansyl (excitation ranges 220–315 and 215–400 nm, resp.; emission ranges 350–500 nm and 425–500 nm, resp.), selecting final conditions of excitation: 278 nm; emission range: 300–520 nm; and scan rate: 1 nm/sec.

Liposomes or proteoliposomes for FRET incorporated dansyl DHPE in chloroform/methanol into HPM lipids in darkness to preserve the fluorescence of the dansyl (final concentration: 2 moL%, based on preliminary experiments comparing 1 and 2 moL%), and vesicles were formed as before. Emission intensity was assessed with 2.0 mL liposome/proteoliposome in a quartz cuvette in a 4-place temperature controlled sample chamber filled with N_2_ gas to minimize any oxidation of protein, which reduces tryptophan fluorescence [30]. Each replicate (*n* = 3) measured fluorescence emission on 4 samples: buffer, liposomes (no protein), ATPase proteoliposomes, and amylase proteoliposomes; all contained 2 moL% dansyl DHPE. Each sample within each replicate was scanned three times within 20 minutes. One additional replicate substituted BSA as the protein in the proteoliposomes.

### 2.7. ATPase Function

The function of Na^+^ K^+^-ATPase was determined (*n* = 4) as the time-dependent release of inorganic phosphate (PO_4_) from ATP (adapted from [31, 32]) in the presence of ATPase proteoliposomes, liposomes (no protein), and also Na^+^ K^+^-ATPase alone (no liposomes present); all samples were prepared as per liposome/proteoliposome methods. The assay was first optimized in 96-well plates for liposome/proteoliposome testing: sample volume (10, 20, 30, 40, 50, 75, and 100 *μ*l/well; *n* = 3), lipid : protein ratio (20 : 1 or 10 : 1; wt : wt; *n* = 4), assay incubation times (20, 30, 40, and 60 minutes; *n* = 4) and optimal ATP concentration (6 mg/mL or 12 mg/mL; *n* = 1). Conditions for the final assay were as follows: proteoliposomes (50.0 *μ*g lipid and 2.25 *μ*g protein/well), lipid-only liposomes (50.0 *μ*g lipid/well), and enzyme-alone (2.25 *μ*g protein/well). The reaction was initiated by the addition of 120 *μ*g ATP/well. Each assay contained a standard curve (0–20 nmoL PO_4_ in duplicate). The assay was incubated (37°C, 60 minutes). The reaction was stopped by addition of a stop reagent (equal parts of 1% (w/v) ammonium molybdate in 12% SDS (w/v in distilled H_2_O) and 6% (w/v) ascorbic acid in 1 N HCl), incubated (5–8 minutes, room temperature), stabilized (2% sodium citrate/2% sodium arsenite/2% acetic acid), and colour allowed to develop (20 minutes, room temperature). Absorbance was then read at 750 nm using a Powerwave X 340 Microplate Scanning Spectrophotometer (Bio-Tek Instruments Inc., Winooski, Vermont, Canada). A control assay included *α*-amylase and BSA proteoliposomes (0.025 mg protein/mL; *n* = 1 for each protein).

### 2.8. ATPase Orientation

The orientation of Na^+^ K^+^-ATPase within the proteoliposomes was assessed with an adapted “sidedness” assay [33]. Sidedness expresses whether the protein is located in the proteoliposome bilayer in one of three orientations: inside-out (*α* subunit facing outwards), right-side-out (*β* subunit facing outwards), or not incorporated (not incorporated into the bilayer). Proteoliposomes were made as before, but in a Na^+^ base buffer (130 mM Na^+^, 4 mM Mg^+2^ and 30 mM histidine, pH 7.0) and identical aliquots were subjected to six treatments (*n* = 3), intact proteoliposomes with no K^+^ **(no K^+^) **± 3 mM ouabain; intact proteoliposomes plus K^+^ ionophores (carbonyl cyanide 3-chlorophenylhydrazone (CCCP) and valinomycin at 1.0 mg/mL and 0.5 mg/mL ethanol resp.) so that K^+^ was on the inside and outside of the proteoliposomes **(K^+^) **± 3 mM ouabain, and liposomes opened with detergent (dodecyloctaethylene glycol monoether (C_12_E_8_), 0.2 mg/mL) in the presence of K^+^ **(open K^+^) **± 3 mM ouabain. The six resulting treatments (Table 1; no K^+^ (B), no K^+^ + ouabain (D), K^+^ (A), K^+^ + ouabain (C), open K^+^ (F), and open K^+^ + ouabain (G)) were then incubated with ATP (60 minutes; 37°C) to measure enzyme activity as before.

Immediately after addition of 0 or 3 mM ouabain, proteoliposomes were provided ATP (final ATP concentration, 3 or 6 mg/mL) and assessed for ATPase function, measured as PO_4_ production (detailed above). Phosphate production was then input into calculations to determine orientation/sidedness as specified by Cornelius [33] based on the treatment groups. Treatment A (K^+^) measures both inside-out and unincorporated Na^+^ K^+^-ATPase, while ouabain (treatment C; K^+^ + ouabain) inhibits this function. Treatments B (no K^+^) and D (no K^+^ + ouabain) are a baseline measure of any background activity. Treatment F (opened) measures total Na^+^ K^+^-ATPase activity in all orientations, while treatment G (opened + ouabain) inhibits all activity and thus measures basal Na^+^ K^+^-ATPase activity. Orientation of Na^+^ K^+^-ATPase is thus determined as [33]:

(1) $$\begin{array}{cc} & \text{Inside}\;\text{out}:\;\frac{\left(\text{A-B}\right)}{\left(\text{F-G}\right)}\;\text{OR}\;\frac{\left(\text{C-D}\right)}{\left(\text{F-G}\right)},\\ & \text{Right}\;\text{side}\;\text{out}:\;\frac{\left(\text{F–A}\right)}{\left(\text{F-G}\right)},\\ & \text{Not}\;\text{incorporated}:\;\frac{\left(\text{B–G}\right)}{\left(\text{F-G}\right)}. \end{array}$$

### 2.9. Effect of Change of Lipid Composition on Na^+^ K^+^-ATPase Orientation

To determine the impact of lipid composition on Na^+^ K^+^-ATPase incorporation and orientation in proteoliposomes, ATPase proteoliposomes were composed of 100% HPM lipids ± 50% select lipids ([34], HPM : SL 1 : 1 (w/w); *n* = 3). SL were a complex mix of various phospholipids with known fatty acid composition (PC : PE : SPH : PS : PI ratio 21 : 26 : 42 : 5 : 5), with a total saturated:unsaturated fatty acid ratio of approximately 85 : 15 [34].

### 2.10. Statistical Analysis

All data were analyzed using SAS 9.1 (SAS 9.1 for Windows TS Level 1M3, The SAS Institute Inc., Cary, NC, USA). Equaliy of variances was accounted for in all data sets.

For the function assay (PO_4_ production), general linear model (GLM) evaluated differences in PO_4_ production among liposomes, proteoliposomes, and Na^+^ K^+^-ATPase. Data were checked for normality and square root transformed if necessary to stabilize the variances. Least squares means (LSM) analysis assessed specific differences where type by method interactions were significant, and contrast statements assessed differences among main effects. Differences between these sample types for lipid : protein ratios of 10: and 20 : 1 were carried out in the same way. *T*-test compared PO_4_ production by proteoliposomes at a lipid : protein of 10 : 1 or 20 : 1 at 60 minutes of incubation. 

A GLM analyzed the FRET raw emission values, determining differences within each replicate using a Tukey's Test. GLM also analyzed the ratios calculated for emission from ATPase proteoliposomes and amylase proteoliposomes expressed relative to the emission intensity of the pure lipid liposomes (set to 100%) within each replicate. Differences were determined using Tukey's Test.

Calculated amounts of Na^+^ K^+^-ATPase in each orientation (inside out, right side out and not incorporated) were analyzed using GLM, and compared by LSM. Overall effect of ouabain was analyzed with GLM, comparing pooled ± ouabain treatments with LSM of log-transformed data.

## 3. Results

### 3.1. Proteoliposome Size

**Figure 1:**
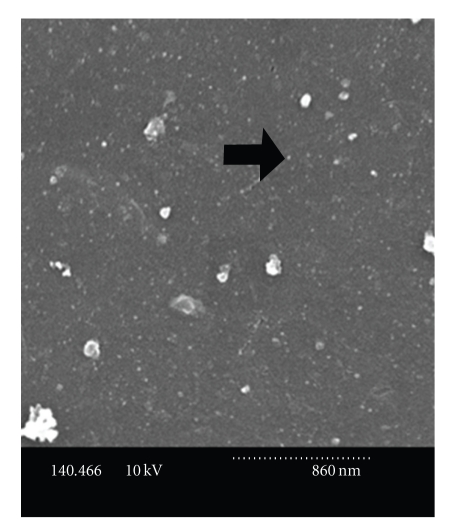
SEM micrograph of typical proteoliposomes. These proteoliposomes were made by high pressure nitrogen filtration, fixed on polished carbon planchettes with OsO_4_, dried, sputter coated with 15 nm argon, and viewed using a Hitachi S-570 Scanning Electron Microscope. The dotted scale bar across the bottom represents 860 nm. Arrow indicates typical liposome.

Dynamic light scattering showed that the diameters of proteoliposomes created using high pressure nitrogen filtration ranged from 58–72 nm and were not statistically different than those of the lipid-only liposomes (54–82 nm). The DLS results were verified with SEM micrographs (Figure 1). 

### 3.2. Protein Incorporation

**Figure 2:**
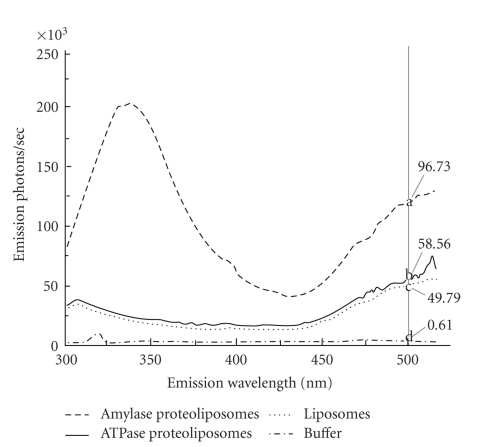
Fluorescence resonance energy transfer to detect ATPase incorporation. Typical fluorescence emission from a single scan of liposomes (dotted) and proteoliposomes containing amylase (dashed) or ATPase (solid) and buffer (dashed dotted). All liposomes were prepared by filtration, contained 2 moL% dansyl, and were excited at 278 nm, the wavelength of excitation for tryptophan endogenous in the enzymes, and emission recorded over 300–520 nm; each preparation was scanned 3 times within 20 minutes. Mean emission intensities obtained from the three scans at 500 nm (grey vertical line), the wavelength of maximum emission of dansyl when excited by emitted light from nearby tryptophan, differed significantly within each replicate (a, b, c, d; *n* = 3).

Both Na^+^ K^+^-ATPase and *α*-amylase contain endogenous tryptophan [12, 35], which was excited during FRET and emitted light at 345 nm, the wavelength of excitation of the fluorophore dansyl which was tagged on the DHPE. The dansyl absorbed this light and the tryptophan-excited dansyl emitted at the empirically measured 500 nm (similar to the published maxima of 497 nm; [36]). BSA proteoliposomes produced negligible emission, consistent with the fact that BSA is a serum protein and should not incorporate into a membrane. Emission intensity differed overall among different vesicles (*P* ≤ .001; Figure 2) and specifically when emission of liposomes with no protein was set to 100% (100 ± 0% < 114.9 ± 2.9% < 161.6 ± 29.3% for liposomes, ATPase, and amylase, respectively; *P* = .052). It is interesting to note that *α*-amylase proteoliposomes had a greater emission at 345 nm, the wavelength of tryptophan emission, than the ATPase liposomes (Figure 2), in accordance with the greater tryptophan content in the amylase compared to ATPase [12, 35].

### 3.3. Proteoliposome Function

**Figure 3:**
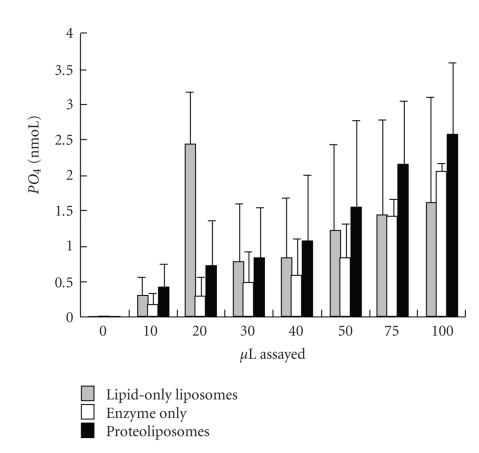
Mean phosphate (PO_4_) detected (nmoL ± SE) in liposomes (lipid-only liposomes), Na^+^ K^+^-ATPase alone (enzyme-only), and proteoliposomes (liposomes containing ATPase) made by filtration (*n* = 3). Measurements were taken at 0, 10, 20, 30, 40, 50, 75, and 100 *μ*l. Liposomes and proteoliposomes contained 0.5 *μ*g lipid/*μ*l prior to vesicle production, while proteoliposomes and enzyme-only contained 0.025 *μ*g Na^+^ K^+^-ATPase/*μ*l. The PO_4_ in each well was detected by reading colour intensity at 750 nm. The slope across concentrations was linear with increasing concentration for enzyme-only and proteoliposomes (*P* < .001).

**Figure 4:**
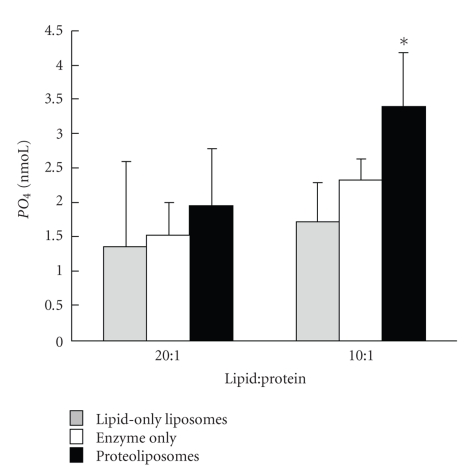
Mean phosphate (PO_4_) detected (nmoL ± SD) in liposomes (lipid-only liposomes), Na^+^ K^+^-ATPase alone (enzyme-only), and proteoliposomes (liposomes containing ATPase) made by filtration with a lipid : protein ratio of 20 : 1 (*n* = 4) or 10 : 1 (*n* = 4) after 60 minutes of incubation. Liposomes and proteoliposomes contained 0.5 *μ*g lipid/*μ*l prior to vesicle production, while proteoliposomes and enzyme-only contained 0.025 *μ*g Na^+^ K^+^-ATPase/*μ*l for 20 : 1 and 0.05 *μ*g Na^+^ K^+^-ATPase/*μ*l for 10 : 1. PO_4_ in each well was detected by reading colour intensity at 750 nm. Significant differences within lipid : protein are marked by ∗.

The Na^+^ K^+^-ATPase incorporated into HPM lipid vesicles was functionally competent to cleave ATP and release PO_4_. Proteoliposomes and enzyme-only produced more PO_4_ with increasing protein concentration in a linear fashion (*P* < .001), while liposomes did not (Figure 3). In data pooled from lipid to protein ratios of 10 : 1 and 20 : 1, proteoliposomes produced more PO_4_ than liposomes and enzyme-only (*P* < .0001). Increasing Na^+^ K^+^-ATPase content increased PO_4_ production overall (10 : 1 lipid : protein > 20 : 1; *P* < .0001) and at 60 minutes of incubation (Figure 4; *P* = .046), but individual earlier time points did not differ significantly (data not shown). Therefore, all subsequent assays were conducted with a 10 : 1 lipid : protein ratio and a 60 minutes incubation time. Proteoliposomes containing BSA or *α*-amylase were much less effective at cleaving PO_4_ than ATPase-proteoliposomes (1.9 and 1.4 versus 7.0 nmoL in a pilot trial). 

### 3.4. ATPase Orientation

**Figure 5:**
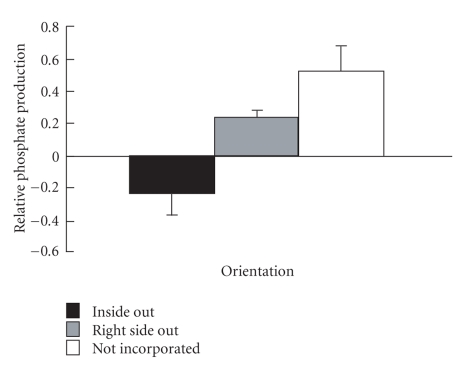
Orientation of Na^+^ K^+^-ATPase in proteoliposomes measured by the sidedness assay (*n* = 3). Proteoliposomes were created by nitrogen filtration, incubated ± ouabain ± detergent and then function (PO_4_ production from ATP) was measured and used to calculate sidedness [33] to determine the relative amount of Na^+^ K^+^-ATPase that was facing inside out, right side out, or was not incorporated. All three orientations of enzyme were statistically different from each other (*P* < .05).

Incubation of Na^+^ K^+^-ATPase-proteoliposomes with the specific inhibitor ouabain lowered PO_4_ production (*P* ≤ .0001). The sidedness assay determined that most Na^+^ K^+^-ATPase in the proteoliposomes was not incorporated into the bilayer (68.3%), but all enzymes that were incorporated were facing right side out (31.7%) and none was inside out (Figure 5). 

### 3.5. Effect of Change of Lipid Composition on Na^+^ K^+^-ATPase Orientation

**Figure 6:**
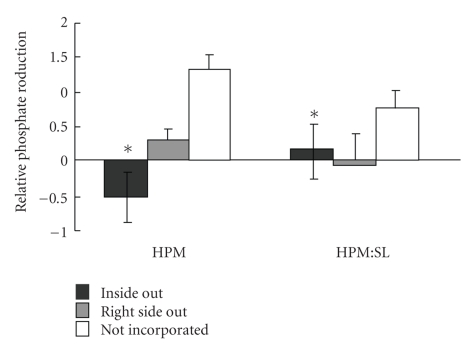
Orientation of Na^+^ K^+^-ATPase in HPM alone and in HPM: SL proteoliposomes measured by the sidedness assay. Proteoliposomes were created by nitrogen filtration using HPM or HPM: SL lipids (1 : 1; wt : wt), incubated ± ouabain ± detergent and then function (PO_4_ production from ATP) was measured and used to calculate sidedness [33] to determine the relative amount of Na^+^ K^+^-ATPase that was facing inside out, right side out, or was not incorporated. Differences between HPM proteoliposomes and HPM: SL proteoliposomes are indicated by ∗.

The orientation of Na^+^ K^+^-ATPase in proteoliposomes made from 100% HPM lipids (as before) differed from that of proteoliposomes made of a mixture of HPM and SL lipids (1 : 1; wt: wt; HPM : SL proteoliposomes), as assessed by the sidedness assay. Although, as before the Na^+^ K^+^-ATPase in both HPM and HPM: SL proteoliposomes was largely not incorporated (84.4%), inclusion of SL lipids caused all ATPase that was incorporated to be inside out (15.6%), and none to be right side out, differing significantly from the exclusively right side out ATPase in the pure HPM liposomes (*P* = .038; Figure 6).

## 4. Discussion

These results convincingly demonstrate that ATPase enzyme can be functionally incorporated into an artificial lipid bilayer and that the lipid composition impacts the orientation and functional ability of amphipathic proteins, consistent with raft theory. 

### 4.1. Proteoliposome Size

The incorporation of Na^+^ K^+^-ATPase into liposomes did not affect the size of small vesicles made by filtration, when compared to lipid-only liposomes. Small, unilamellar proteoliposomes, such as those produced by high pressure filtration methods established here, are therefore appropriate for the study of protein function [37].

### 4.2. Protein Incorporation

A measurement of the proximity of lipid and protein molecules in relation to each other using fluorescence resonance energy transfer can be employed to determine if a protein is incorporated into a lipid bilayer. FRET uses the energy from one molecule, the donor, here the tryptophan present in Na^+^ K^+^-ATPase or *α*-amylase (see [12, 35], resp.), to emit light at a wavelength (tryptophan, 345 nm) that excites a second acceptor molecule, here dansyl. Dansyl's resultant emission was then detected at 497 nm [36]. The key is that the energy transfer can only occur over very short distance, so the donor and acceptor must be in close enough physical proximity to interact in this way. If they are not, emission from the donor molecule will not be absorbed by the acceptor and thereby excite it: the unabsorbed light emitted by the donor will therefore be emitted [36].

 Optimal conditions were first established to detect resonance energy transfer, using 2 moL% dansyl (energy acceptor), a lipid : protein ratio of 10 : 1 (HPM: Na^+^ K^+^-ATPase) which also was the optimum lipid : protein ratio to measure ATPase function. This is similar to the lipid : protein ratios commonly used for Na^+^ K^+^-ATPase function studies [38]. Our identified nonoxidizing N_2_ atmosphere parallels the need because oxidation of tryptophan decreases its fluorescence [30]. 

Emission from ATPase proteoliposomes was consistently significantly higher than in liposomes alone, indicating that ATPase was incorporated into the lipid bilayer and its tryptophan excited. Amylase proteoliposomes elicited significantly higher dansyl emission than ATPase proteoliposomes because of the approximate 2-fold greater number of tryptophan moieties in *α* amylase compared to Na^+^ K^+^-ATPase [12, 35]. The *α*-amylase proteoliposomes showed a large peak at the tryptophan emission wavelength, which both corresponds with the reported high level of tryptophan present and suggests that the tryptophan emission exceeded the absorbance capacity of the dansyl molecules in the membrane.

### 4.3. Proteoliposome Function

Functionality of ATPase in liposomes was assessed by measuring the enzymatic ability to cleave phosphate from ATP. Proteoliposomes containing Na^+^ K^+^-ATPase produced significantly more PO_4_ than BSA or *α*-amylase proteoliposomes, or liposomes alone, indicating that the incorporated Na^+^ K^+^-ATPase enzyme is indeed functional under the optimal assay conditions of 10 : 1 lipid : protein incubated for 60 minutes incubation time, which is in the range used by others [23].

### 4.4. Orientation of Na^+^ K^+^-ATPase in HPM Liposomes

Having demonstrated a reliable method to produce proteoliposomes with functional incorporated ATPase in the bilayer, it was important to know the orientation of the enzyme. Certainly the lipids in sperm head plasma membranes are not randomly distributed [39–41]. Furthermore, since HPM lipids from boar sperm form distinct domains of differing fluidities, which change over time, or with cryopreservation and/or with the medium in which sperm are preserved [42], the HPM lipids in the liposomes here were expected to similarly organize in a nonrandom manner. If the Na^+^K^+^-ATPase preferentially interacted with certain lipids, its orientation should be nonrandom, which was exactly what the sidedness assay indicated. All the enzyme that was incorporated, was incorporated in a right-side-out orientation (*β* subunit facing outwards). Others have found that the sidedness of Na^+^ K^+^-ATPase in artificial membrane differs depending on the source of protein, the method of reconstitution, and especially on the lipid composition of the vesicles [23]. Vesicles of PC made by detergent dialysis caused 50% of ATPase to orient inside out [43], while in liposomes made of PE, PC, PI, and cholesterol by Bio Bead detergent removal, the ratio for Na^+^ K^+^-ATPase right side out: inside out: not oriented was 65 : 15 : 20 [44]. The liposomes here made by nitrogen filtration with native sperm HPM lipids allowed effective incorporation of functional Na^+^ K^+^-ATPase, albeit at a low level.

### 4.5. Effect of Lipid Composition on Orientation of Na^+^ K^+^-ATPase

Altering the lipid composition of the vesicles did not affect the amount of protein that was not incorporated but did affect the orientation of Na^+^ K^+^-ATPase in the membrane. When the bilayers contained endogenous native lipids, all the ATPase was oriented with its *β* subunit in the outer leaflet, but replacing the HPM with 50% selected mixed lipids significantly increased the amount of Na^+^ K^+^-ATPase oriented with its *β* subunit in the inner leaflet. 

Na^+^ K^+^-ATPase orientation in reconstituted vesicles is affected by lipid composition, more specifically by the acyl chain lengths of phospholipids [45], the degree of saturation of the phospholipids [46], and the amount of cholesterol present [47, 48]. Acyl chain lengths of phospholipids are important for determining the thickness of membrane bilayers. Bilayers that are too thick or too thin to accommodate the hydrophobic region of Na^+^ K^+^-ATPase may result in improper folding, altered function, and differing levels of inclusion of ATPase [49]. Increasing the acyl chain length of monounsaturated PC results in an increase in the amount of Na^+^ K^+^-ATPase that is not incorporated into liposomes, and the optimal amount of protein incorporated into liposomes occurs at 14- and 20-carbon chain length for unsaturated and saturated PC, respectively [50]. Cholesterol inclusion works to increase the thickness and order of plasma membranes [51] and it has been suggested that some membrane domains are formed only in the presence of certain concentrations of cholesterol [52]. Lipid rafts are enriched in cholesterol, sphingomyelin, lipids with saturated long chain acyl chains, [1] and in sperm, the plasma membrane which lays over the acrosome displays rafts enriched in gangliosides and cholesterol [53, 54]. Cholesterol content of membranes both above and below native membrane content (16 moL% of total lipids) decreases Na^+^ K^+^-ATPase activity in PC liposomes using pig kidney ATPase [47]. While the addition of 40 moL% cholesterol into PC liposomes had no effect on the amount of Na^+^ K^+^-ATPase that was not incorporated into liposomes, it doubled the amount of enzyme that was inside out (right-side-out enzyme was not measured; [50]). 

The extracted HPM lipids were measured and consisted of 45.2% PC, 9.7% PE, 5.7% Sph, 5.4% PI, 4.2% PS, and 29.8% cholesterol, with 29.3% of the total phospholipids being saturated. Most saturated phospholipids were 16 carbons long, while the majority of the unsaturated phospholipids had 22 carbon acyl chains. Therefore liposomes made with 50% HPM : 50% selected lipids would have increased quantities of Sph (16 : 0 and 18 : 0) and PE (18 : 0), along with moderate increases in other phospholipids. The amount of overall cholesterol would be decreased by 50%. Although reducing the cholesterol did not affect the amount of ATPase incorporated into the liposomes, either here or for Cornelius [50], the lower cholesterol content could have caused the altered orientation, since removal of cholesterol from sperm membranes alters raft structure, changing the size, distribution, and content of glycoproteins [54–56]. It would be interesting to test ATPase orientation in liposomes with systematically altered cholesterol content.

Lipid rafts have been variously suggested to break down [57] or become enriched [3, 54] during sperm capacitation. Regardless of how rafts change, it is clear that the lipid rafts in sperm membranes change during capacitation. Seminolipid, a sperm-specific glycolipid concentrated in the outer leaflet of the plasma membrane on the apical ridge of the sperm head [56, 58], may have the ability to stabilize the plasma membrane, preventing the acrosome reaction [56, 59]. Depleting cholesterol from the plasma membrane during capacitation facilitates the removal of seminolipid [60]. Consistent with activation of Na^+^ K^+^-ATPase by raft-mediated relocation in other cells [19, 20], ATPase in sperm may be relocated in the plasma membrane by the efflux of cholesterol during capacitation so that it is accessible to bind ouabain and initiate signalling cascade. Cholesterol efflux from sperm membranes during capacitation occurs more readily in nonraft or fluid fractions of the membrane [54]. Certainly the current results demonstrate that Na^+^ K^+^-ATPase is sensitive to its lipid environment, and that lipid environment alters its orientation, and therefore its accessibility to external stimuli such as ouabain.

## 5. Conclusions

Functional Na^+^ K^+^-ATPase can be incorporated into proteoliposomes. High pressure nitrogen filtration applied to native lipids extracted from sperm membranes reliably creates consistently sized liposomes and proteoliposomes. FRET elegantly proves that the proteoliposomes have actually incorporated the amylase or ATPase proteins into the lipid bilayer. Incorporated ATPase is functional and, in a bilayer of native lipids, is oriented exclusively in a right side-out orientation. Alteration of the lipid composition changes the orientation of Na^+^ K^+^-ATPase in the lipid bilayer, confirming that specific lipids effectively influence integral membrane proteins. These results support recent results suggesting that inclusion of raft-associated lipids and proteins may increase the quantity and stabilize the orientation of Na^+^ K^+^-ATPase in model, or cellular, membranes.

## Figures and Tables

**Table 1:** Outline of treatments included in the sidedness assay. One batch of proteoliposomes was prepared and split into 6 × 400 *μ*l aliquots, each aliquot preincubated (2 minutes, room temperature) with reagents indicated, then 0 or 3 mM ouabain added, and samples incubated with ATP (60 minutes; 37°C). Adapted from [33].

Treatment		no K^+^		K^+^		Opened	
		B	D	A	C	F	G
Pre incubation	K^+^	0	0	yes	yes	yes	yes
	Ionophores	0	0	yes	yes	yes	yes
	Detergent	0	0	0	0	yes	yes

Post incubation	Ouabain	0	yes	0	yes	0	yes
